# Neuroinflammation at the Dorsolateral Inferior Medulla: A Possible Central Nervous System Localization for POTS and Long COVID

**DOI:** 10.3390/biomedicines13010166

**Published:** 2025-01-12

**Authors:** Svetlana Blitshteyn

**Affiliations:** 1 Department of Neurology, University of Buffalo Jacobs School of Medicine and Biomedical Sciences, Buffalo, NY 14203, USA; 2 Dysautonomia Clinic, Williamsville, NY 14221, USA; sb25@buffalo.edu; Tel.: +1-716-531-4598; Fax: +1-716-478-6917

**Keywords:** long COVID, postural orthostatic tachycardia syndrome, brainstem, medulla, neuroinflammation

## Abstract

Both postural orthostatic tachycardia syndrome (POTS) and Long COVID are currently viewed as heterogeneous disorders with complex, multi-factorial and multi-systemic pathophysiology. POTS, one of the most common autonomic disorders, is a frequent sequela of SARS-CoV-2 infection. Both POTS and autonomic dysfunction, in general, are major pathophysiologic mechanisms of Long COVID. There is emerging evidence that neuroinflammation of the brainstem may be one of the mechanisms of POTS and Long COVID. This commentary argues that neuroinflammation at the dorsolateral inferior medulla is a possible central nervous system localization for POTS and Long COVID based on the limited scientific literature available to date and the neurologic manifestations of both disorders. Further studies involving advanced neuroimaging techniques and animal models with immunohistochemical brainstem tissue assessments are needed to understand how and why possible neuroinflammation at the dorsolateral inferior medulla may occur in patients with Long COVID, POTS and other disorders involving autonomic dysfunction.

## 1. Introduction

Over the last 30 years since postural orthostatic tachycardia syndrome (POTS) was defined in the scientific literature, numerous mechanistic factors have been identified as possible pathophysiology, including the involvement of the central nervous system (CNS). POTS, one of the most common disorders of the autonomic nervous system (ANS), is now an accepted frequent sequela of SARS-CoV-2 infection, and autonomic dysfunction more broadly is one of the major components of Long COVID [1,2,3].

POTS is defined by the following diagnostic criteria: A sustained heart rate elevation of at least 30 bpm in adults and at least 40 bpm in teens 12–19 years of age from supine to standing position during a 10 min stand test or a tilt table test; the absence of orthostatic hypotension; symptoms of orthostatic intolerance must be present for at least 3 months [4]. POTS is characterized by orthostatic tachycardia and orthostatic intolerance, but CNS symptoms, such as headache, fatigue, chronic dizziness, cognitive dysfunction and sleep disturbance, affect most patients. These symptoms cause significant functional impairment and are typically less amenable to treatment than the hallmark feature of postural tachycardia.

## 2. POTS, Long COVID and the CNS

POTS is commonly viewed as a syndrome affecting the peripheral nervous system via autonomic nerve fibers, with 50% of patients having small fiber neuropathy [5]. However, CNS pathophysiology with ANS dysfunction and brainstem dysregulation has been suggested as a possible mechanism of POTS before the COVID-19 pandemic [6,7]. The ANS consists of peripheral and central autonomic pathways, with the central input originating from the hypothalamus and descending through the nuclei in the rostral medulla and caudal pons to peripheral target organs via the sympathetic nervous system and parasympathetic nervous system, which, in turn, send sensory signals back to the brain [7]. The limbic system, amygdala and insular cortex are important central regions that are involved in regulating emotions, behavior and homeostasis utilizing the ANS [8]. The dorsal medulla is the main location of the autonomic nuclei responsible for blood pressure regulation and integration of the vagal afferent and efferent pathways [8].

Pre-pandemic, POTS was known to occur after a viral or bacterial infection in at least 40% of patients and was thought to be of heterogeneous and complex pathophysiology involving central and peripheral autonomic nervous system networks, small fiber neuropathy, cerebral hypoperfusion, mast cell hyperactivity and autoimmunity [9,10,11]. In fact, given its multitude of CNS symptoms and a positive response to CNS-penetrating medications, including stimulants, in many patients, POTS has been considered to be a possible CNS disorder [6], but studies on neuroimaging and CNS pathophysiology in POTS have been extremely limited. One study before the COVID-19 pandemic, using (^1^H) magnetic resonance spectroscopy to quantify markers of neuronal and glial integrity, demonstrated evidence of neuroinflammation at the dorsal medulla in pediatric patients with orthostatic intolerance vs. healthy controls [12]. In their study, the dorsal medulla was hypothesized to be an important locus and area of interest in patients with orthostatic intolerance and postural tachycardia—a potential localization that made sense because the nuclei of the vagus nerve were located in this area [12].

With the renewed interest in POTS and autonomic dysfunction as a result of the SARS-CoV-2 pandemic, we are now able to elucidate more precisely and confirm the pre-pandemic hypothesis that POTS is a central nervous system disorder affecting the dorsolateral inferior medulla [6]. Recently, in an innovative and informative study utilizing ultra-high field (7T) quantitative susceptibility mapping of the brain in patients with post-COVID symptoms after hospitalization, Rua et al. identified increased MR susceptibility in the medulla, pons and midbrain areas of the brainstem, specifically in the reticular formation and raphe areas of the inferior medulla [13]. Their results suggest neuroinflammation in these regions though further studies of a large cohort are necessary to confirm these findings. While the study participants were not specifically tested for POTS, autonomic dysfunction is one of the major mechanisms of lingering symptoms after SARS-CoV-2, in both hospitalized patients and those with mild infection that necessitates its own diagnostic and therapeutic approaches [2,3]. In the National Academies of Science definition of Long COVID, autonomic dysfunction, including POTS—one of the most common autonomic disorders—is listed as a major feature of Long COVID [3]. Although the mechanisms of Long COVID have not been fully delineated, immunologic, autoimmune, endothelial, autoimmune and hypercoagulable alterations have been identified [3,14]. We can, therefore, consider findings from studies on patients with Long COVID to be relevant to patients with POTS or at least 40% of patients with POTS whose illness began after an infection.

## 3. Dorsolateral Medulla: Localization for POTS and Long COVID

Dorsolateral inferior medulla as a possible localization for Long COVID, post-COVID dysautonomia and POTS, in general, is a reasonable consideration from the standpoint of clinical–anatomical correlation [6,12,13]. First, the reticular formation serves as a relay between the peripheral and central nervous system in how the brainstem controls cardiopulmonary functions, respiration and consciousness. The reticular formation is vital to numerous critical functions, including arousal, consciousness, circadian rhythm, sleep–wake cycles, coordination of somatic motor movements, cardiovascular and respiratory control, pain modulation and habituation [15]. The vasomotor center present in the medulla modulates cardiovascular control. The central areas, located caudally in the reticular formation at the pontomedullary junction, play a role in the autonomic rhythms of respiration. These central areas are also connected to the cranial nerve motor nuclei of the trigeminal, facial, glossopharyngeal, vagus and hypoglossal nerves to result in the coordinated function of respiration [15].

**Figure 1:**
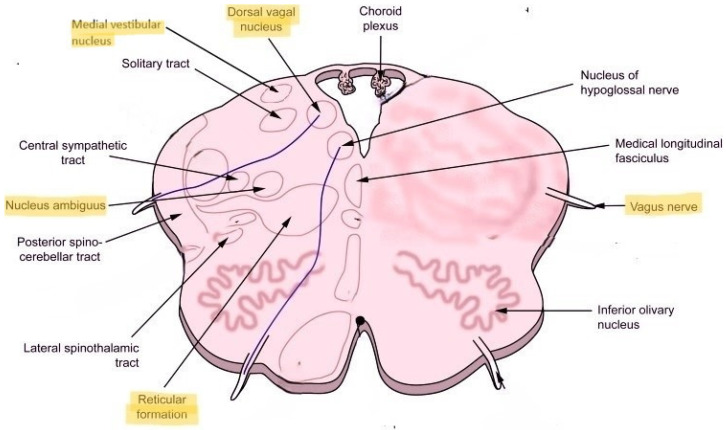
Cross-section of the inferior medulla with highlighted structures that may form the central nervous system localization of POTS and Long COVID.

Second, the nucleus ambiguus, which carries some of the preganglionic parasympathetic fibers, is located proximal to the reticular formation (Figure 1). The vagus nerve and some of its fibers that come out of the nucleus ambiguus carry the parasympathetic nerve fibers and innervate the heart and lungs. Disturbance of the parasympathetic outflow via the vagus nerve may result in reduced heart rate variability and respiration control underlying autonomic dysfunction. Located more posteriorly is the dorsal nucleus of the vagus nerve, which is a major carrier of the parasympathetic output to the heart and lungs. Located more laterally is the medial vestibular nucleus, which is important in the control of head, neck and body position as well as equilibrium and balance (Figure 1). Since Long COVID and POTS commonly manifest with dizziness, fatigue, headache and blood pressure, heart rate and temperature dysregulation, localization to the dorsolateral medulla with possible neuroinflammation involving nucleus ambiguus, dorsal nucleus of the vagus and medial vestibular nucleus seems plausible from the standpoint of clinical–anatomical correlation.

Finally, Rua et al. specifically name nuclei raphe pallidus and obscurus as having increased susceptibility on 7T MRI in their study [13]. These nuclei appear to be highly relevant to the central autonomic networks and brainstem-controlled immunologic response. Specifically, the nucleus raphe pallidus is known to mediate the tachycardia response and appears to be involved in the activation of a fever as an immunoreaction [16]. Tachycardia is a defining feature of POTS, and both tachycardia and recurrent fever are common manifestations of Long COVID and other post-acute infectious syndromes.

**Figure 2:**
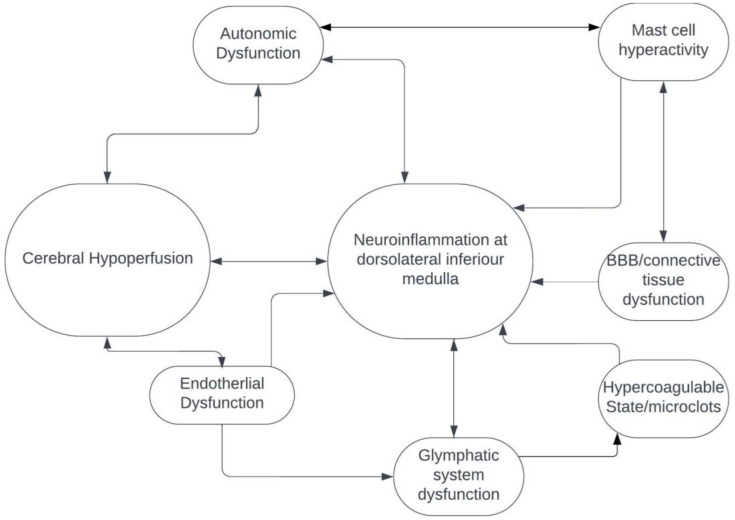
Mechanisms that may lead to neuroinflammation at the dorsolateral inferior medulla.

Cerebral hypoperfusion is one of the major mechanisms of POTS, autonomic dysfunction, myalgic encephalomyelitis/chronic fatigue syndrome and probably Long COVID [6]. How cerebral—and, in particular, brainstem—hypoperfusion fits with neuroinflammation of the dorsolateral inferior medulla is currently unknown and needs to be investigated. It is possible that small penetrating arteries coming off the vertebrobasilar arterial system and supplying the brainstem may be damaged from endothelial dysfunction, hypercoagulable state and possible microclots, all of which appear to be important in the pathogenesis of Long COVID (Figure 2) [14]. Additionally, and/or alternatively, alterations in the glymphatic system may result in inadequate venous drainage of the dorsolateral medulla, venous congestion and toxin accumulation, further leading to neuroinflammation (Figure 2) [17]. Finally, abnormalities in the connective tissue itself, possibly resulting from neuroinflammatory and immune-mediated pathways that include hyperactivated mast cells, could alter the composition of venous and arteriolar walls and the blood–brain barrier, which could, in turn, result in abnormal vascular contractility and stiffening, thereby causing decreased perfusion of the brainstem (Figure 2) [14,17]. Importantly, further studies are needed to elucidate the interplay of these and other possible identified and unidentified mechanisms that can induce neuroinflammation of the brainstem, which, in turn, may lead to a wide range of diverse neurologic and non-neurologic symptoms and signs of post-acute infectious syndromes.

## 4. Future Direction

More questions and uncertainties regarding CNS localization and pathophysiology remain, including the following:

- Why would neuroinflammation affect only specific areas of the brainstem and not the rest of the brainstem or the subcortical and cortical regions in POTS and Long COVID?
- How do hypovolemia, small fiber neuropathy and autoimmunity factor in the development of neuroinflammation at the dorsolateral medulla?
- Is neuroinflammation at the dorsolateral medulla the cause or consequence of systemic immune dysregulation?
- Might there be a mechanical brainstem compression at the level of the dorsolateral inferior medulla with resultant secondary neuroinflammation?
- Could there be structural changes in the ligaments, bones and glymphatic system at the cranio-cervical junction, which may be caused by connective tissue alterations that are triggered by acute infection, possibly in a host with a genetic predisposition toward hypermobility spectrum disorders and other aberrant connective tissue phenotypes?
- How do hyperactive mast cells triggered by infection cause or contribute to neuroinflammation, and do antihistamines and mast cell stabilizing agents reduce neuroinflammation?
- How can we develop clinically available and affordable diagnostic biomarkers to objectively confirm brainstem neuroinflammation when 7T MRI is not available for clinical testing at this time and 3T MRI fails to detect the radiographic signs of neuroinflammation?
- Ultimately, the most important question is the following: What therapeutic targets should be explored in patients with POTS and Long COVID?

It appears that immunomodulating therapies might be best suited to address the inflammatory and hyperimmune process driving post-acute infectious syndromes, but other treatment options may include antivirals, parasympathetic nervous system enhancers including non-invasive vagus nerve stimulation, sympatholytics, blood volume expanders, endothelial dysfunction modulators, vasoconstrictors and vasodilators that alter vascular tone, antiplatelet and anticoagulant therapies, antihistamine and mast cell stabilizing agents that decrease mast cell and sympathetic overactivity and GLP-1 receptor agonists that may reduce neuroinflammation. Other possible treatment options that may deserve further consideration are transcranial magnetic stimulation, hyperbaric oxygen therapy and cerebral venous stenting in patients with venous stenosis or congestion that may be causing or contributing to neuroinflammation.

Finally, a question that needs to be answered is whether the available therapies for other neuroinflammatory and neuroimmune CNS disorders, such as multiple sclerosis, neurologic Sjogren’s, neuropsychiatric lupus, CNS vasculitis and others, can be beneficial for patients with POTS, Long COVID and other post-acute infectious syndromes with neuropsychiatric manifestations. Currently, there are no accepted practices, recommendations or guidelines for utilizing existing immunologic therapies in patients with brain or brainstem dysfunction and neuropsychiatric manifestations of post-acute infectious syndrome, rendering these therapies inaccessible to patients. Thus, there is a need to examine this topic thoroughly and urgently in light of the ongoing COVID-19 pandemic and post-acute sequelae of SARS-CoV-2. Repurposed therapies might offer a safe, effective and rapid treatment pathway toward improvement and possible recovery for many millions of patients with Long COVID and POTS.

## 5. Conclusions

In summary, Rua et al. identified evidence of neuroinflammation using 7T MRI in previously hospitalized patients with post-COVID symptoms at specific regions of the brainstem that are critical to the autonomic nervous system control of the organs and vital physiologic processes, including respiration, heart rate, blood flow, digestive system, bladder function and probably immunologic response [13]. The highly diverse and multisystemic symptoms displayed by most patients with Long COVID correlate with dysfunction and structural abnormalities seen on 7T MRI of the dorsolateral inferior medulla, which may also be the localization for POTS and autonomic dysfunction more broadly, unrelated to SARS-CoV-2 infection. Further studies involving advanced neuroimaging techniques and animal models with immunohistochemical brainstem tissue assessments are needed to understand how and why possible neuroinflammation at the dorsolateral inferior medulla may occur in patients with Long COVID, POTS and other disorders involving autonomic dysfunction. Already preliminary evidence suggests that neuroinflammation at the dorsolateral inferior medulla is a possible central nervous system localization for POTS and Long COVID [6,12,13].

## References

[1] Blitshteyn S., Whitelaw S. (2021). Postural orthostatic tachycardia syndrome (POTS) and other autonomic disorders after COVID-19 infection: A case series of 20 patients. Immunol. Res..

[2] Blitshteyn S., Whiteson J.H., Abramoff B., Azola A., Bartels M.N., Bhavaraju-Sanka R., Chung T., Fleming T.K., Henning E., Miglis M.G. (2022). Multi-disciplinary collaborative consensus guidance statement on the assessment and treatment of autonomic dysfunction in patients with post-acute sequelae of SARS-CoV-2 infection (PASC). PM R.

[3] National Academies of Sciences, Engineering, and Medicine (2024). A Long COVID Definition: A Chronic, Systemic Disease State with Profound Consequences.

[4] Sheldon R.S., Grubb B.P., Olshansky B., Shen W.K., Calkins H., Brignole M., Raj S.R., Krahn A.D., Morillo C.A., Stewart J.M. (2015). 2015 heart rhythm society expert consensus statement on the diagnosis and treatment of postural tachycardia syndrome, inappropriate sinus tachycardia, and vasovagal syncope. Heart Rhythm.

[5] Peltier A.C., Garland A., Raj S.R. (2010). Distal sudomotor findings in postural tachycardia syndrome. Clin. Auton. Res..

[6] Blitshteyn S. (2022). Is postural orthostatic tachycardia syndrome (POTS) a central nervous system disorder?. J. Neurol..

[7] Novak V., Novak P., Opfer-Gehrking T.L., O’Brien P.C., Low P.A. (1998). Clinical and laboratory indices that enhance the diagnosis of postural tachycardia syndrome. Mayo Clin. Proc..

[8] Benarroch E. (1993). The central autonomic network: Functional organization, dysfunction, and perspective. Mayo Clin. Proc..

[9] Shaw B.H., Stiles L.E., Bourne K., Green E.A., Shibao C.A., Okamoto L.E., Garland E.M., Gamboa A., Diedrich A., Raj V. (2019). The face of postural tachycardia syndrome-insights from a large cross-sectional online community-based survey. J. Intern. Med..

[10] Boris J.R., Shadiack EC 3rd McCormick E.M., MacMullen L., George-Sankoh I., Falk M.J. (2024). Long-term POTS outcomes survey: Diagnosis, therapy, and clinical outcomes. J. Am. Heart Assoc..

[11] Grubb A.F., Grubb B.P. (2023). Postural orthostatic tachycardia syndrome: New concepts in pathophysiology and management. Trends Cardiovasc. Med..

[12] Wagoner A.L., Olson J.D., Westwood B.M., Fortunato J.E., Diz D.I., Shaltout H.A. (2019). Children with orthostatic intolerance exhibit elevated markers of inflammation in the dorsal medulla. Am. J. Physiol. Heart Circ. Physiol..

[13] Rua C., Raman B., Rodgers C.T., Newcombe V.F.J., Manktelow A., A Chatfield D., Sawcer S.J., Outtrim J.G., Lupson V.C., A Stamatakis E. (2024). Quantitative susceptibility mapping at 7 T in COVID-19: Brainstem effects and outcome associations. Brain.

[14] Turner S., Khan M.A., Putrino D., Woodcock A., Kell D.B., Pretorius E. (2023). Long COVID: Pathophysiological factors and abnormalities of coagulation. Trends Endocrinol. Metab..

[15] Mangold S.A., Das J.M. (2024). Neuroanatomy, Reticular Formation. StatPearls.

[16] Nakamura K., Matsumura K., Kaneko T., Kobayashi S., Katoh H., Negishi M. (2002). The rostral raphe pallidus nucleus mediates pyrogenic transmission from the preoptic area. J. Neurosci..

[17] Wu L., Zhang Z., Liang X., Wang Y., Cao Y., Li M., Zhou F. (2023). Glymphatic system dysfunction in recovered patients with mild COVID-19: A DTI-ALPS study. iScience.

